# Aquaporin‐1 inhibition reduces metastatic formation in a mouse model of melanoma

**DOI:** 10.1111/jcmm.13378

**Published:** 2017-10-17

**Authors:** Laura Simone, Concetta Domenica Gargano, Francesco Pisani, Antonio Cibelli, Maria Grazia Mola, Antonio Frigeri, Maria Svelto, Grazia Paola Nicchia

**Affiliations:** ^1^ IRCCS Casa Sollievo della Sofferenza Institute for Stem Cell Biology Regenerative Medicine and Innovative Therapies (ISBReMIT) San Giovanni Rotondo FG Italy; ^2^ Department of Bioscience Biotechnologies and Biopharmaceutics Centre of Excellence in Comparative Genomics University of Bari “Aldo Moro” Bari BA Italy; ^3^ School of Medicine Basic Medical Sciences Neuroscience and Sense Organs University of Bari “Aldo Moro” Bari BA Italy; ^4^ Department of Neuroscience Albert Einstein College of Medicine Yeshiva University, New York Bronx NY USA; ^5^ Institute of Biomembranes and Bioenergetics National Research Council Bari BA Italy; ^6^ National Institute of Biostructures and Biosystems (INBB) Rome Italy

**Keywords:** aquaporin‐1, tumour angiogenesis, metastasis, melanoma, antiangiogenic therapy, endothelial migration, apoptosis, extracellular matrix

## Abstract

Aquaporin‐1 (AQP1) is a proangiogenic water channel protein promoting endothelial cell migration. We previously reported that AQP1 silencing by RNA interference reduces angiogenesis‐dependent primary tumour growth in a mouse model of melanoma. In this study, we tested the hypothesis that AQP1 inhibition also affects animal survival and lung nodule formation. Melanoma was induced by injecting B16F10 cells into the back of C57BL6J mice. Intratumoural injection of AQP1 siRNA and CTRL siRNA was performed 10 days after tumour cell implantation. Lung nodule formation was analysed after the death of the mice. Western blot was used to quantify HIF‐1α, caspase‐3 (CASP3) and metalloproteinase‐2 (MMP2) protein levels. We found that AQP1 knock‐down (KD) strongly inhibited metastatic lung nodule formation. Moreover, AQP1 siRNA‐treated mice showed a twofold survival advantage compared to mice receiving CTRL siRNAs. The reduced AQP1‐dependent tumour angiogenesis caused a hypoxic condition, evaluated by HIF‐1α significant increase, in turn causing an increased level of apoptosis in AQP1 KD tumours, assessed by CASP3 quantification and DNA fragmentation. Importantly, a decreased level of MMP2 after AQP1 KD indicated a decreased activity against extracellular matrix associated with reduced vascularization and metastatic formation. In conclusion, these findings highlight an additional role for AQP1 as an important determinant of tumour dissemination by facilitating tumour cell extravasation and metastatic formation. This study adds knowledge on the role played by AQP1 in tumour biology and supports the view of AQP1 as a potential drug target for cancer therapy.

## Introduction

Aquaporins (AQPs) are transmembrane channel proteins responsible for water flux across the plasma membrane driven by osmotic and hydrostatic gradient 1. Predictably, AQPs sustain physiological mechanisms associated with a large movement of fluids, such as the urinary concentration in the kidney 2, 3. However, AQPs also play a key role in facilitating changes in cell shape and volume occurring due to local alteration of the osmotic balance between the inside and the outside of the cell 4, 5, 6. Cell migration and cell swelling or shrinkage, for instance, are mechanisms occurring through such changes in shape and volume and sustained by cytoskeleton dynamics 7, 8. Studies performed with AQP null mice have revealed an important role of aquaporin‐4 (AQP4) for the migration of astrocytes forming the glial scar in the central nervous system 9 and of aquaporin‐1 (AQP1) for the migration of endothelial cells in tumour angiogenesis 10, 11. AQPs would positively modulate cell migration by facilitating the osmotic water influx at the leading edge of the cells in response to a local osmotic gradient created by active solute influx 12, 13. Based on their role in cell volume regulation and cell migration, AQPs, and in particular the endothelial water channel AQP1 play an important role in tumour biology 14. Available literature shows increased AQP1 expression in aggressive tumours such as colon, breast, lung, liver cancer and brain tumours 15, 16, 17, 18, 19, 20, 21. Importantly, AQP1 expression increases in proliferating tumour microvessels in human 22, 23, 24, and studies performed with AQP null mice have revealed that AQP1 deletion strongly impairs the migration of endothelial cells in tumour angiogenesis in mouse model of melanoma 11.

Tumour angiogenesis is the formation of new blood vessels necessary to provide nutrients and oxygen for tumour to growth. The progression of the migrating endothelial cells requires the degradation of the extracellular matrix mediated by matrix metalloproteinase (MMPs), a family of zinc‐dependent endopeptidases. MMPs are secreted by cells resident in the tumour, such as tumour cells, fibroblasts and activated endothelial cells, and mainly by immigrating immune cells 25. Among MMPs, metalloproteinase‐2 (MMP2) is involved in mechanisms, such as tumour invasion and tissue infiltration of T lymphocytes, in which a disruption of the basement membrane is required 26. The new vessel architecture is irregular and characterized by a very high permeability, therefore representing an ideal route by which cancer cells can escape the tumour and disseminate within the body to form metastasis 27. The vascular endothelial growth factor (VEGF) pathway is up to now the main target for antiangiogenic therapy. However, resistance and adaptation of cancer patients and, in general, the lack of the expected benefits of anti‐VEGF therapies have underlined the need to focus on a next generation of antiangiogenic drugs based on pathways alternative to VEGF 28.

The relationship between AQP1 expression and metastatic potential and survival has been previously shown in invading lung cancer, particularly in adenocarcinomas, where AQP1 overexpression correlated with metastases and unfavourable survival rates 29. In a previous work, we reported that AQP1 silencing impairs AQP1‐dependent tumour angiogenesis and tumour growth in a mouse model of melanoma 30. These scenarios prompted us to investigate the mechanistic correlation between AQP1, animal survival and formation of metastases. Metastasis formation is often studied by injecting B16 cell line endovenously into C57BL6/J mice, as they are able to root in the lungs and form nodules. However, using this model, the key tumour cell intravasation step of the metastatic process is bypassed. Therefore, we have here used the same syngenic mouse model of tumour used in our previous study 30 in which B16F10 cells are injected into the flank of C57BL6/J mice where they form the primary tumour. Among the B16 cell line, the B16F10 subclone is characterized by the highest metastatic potential; therefore, metastases arise in the lungs from the primary tumour 31, 32.

We report here the proof of concept that AQP1 inhibition is also able to strongly reduce cell's ability to metastasize by crossing endothelial barrier and further support AQP1 as proangiogenic protein and potential target for cancer therapy.

## Materials and methods

### Ethics statement

Procedures involving animals were carried out in compliance with the European directive on animal use for research and Italian law on animal care. This project has been approved by the Italian Health Department (Approved Project no. 100/2014‐B). All experiments were designed to minimize the number of animals used and animal suffering.

### Cell culture

The murine melanoma B16F10 cells CRL 6475 (www.lgcstandards-atcc.org) were grown in DMEM medium supplemented with 10% heat‐inactivated foetal bovine serum (FBS), 100 U/ml penicillin and 100 mg/ml streptomycin and maintained at 37°C in a 5% CO_2_ incubator. Cell culture reagents were purchased from Thermofisher (www.thermofisher.com). In experiments using staurosporine (www.sigmaaldrich.com), cells were cultured for 24 hrs, treated with drugs for 48 hrs and then collected.

### Short interfering stealth RNAs

AQP1 siRNA (5′‐ACUCCAGGGUGGAGAUGAAGCCCAA‐3′) and a scrambled CTRL siRNA (5′‐CGAUGGAGAAGGCCAACUAGGGACU‐3′) 30 specific for mouse were purchased from Thermo Fisher Scientific (www.thermofisher.com).

### Implantation of B16F10 tumour cells and RNAi experiments in mice

Male C57BL6/J mice (8 weeks of age) were obtained from Harlan Laboratories (www.envigo.com). Animals were kept in a regulated environment (23 ± 1°C, 50 ± 5% humidity) with a 12 hrs light/dark cycle with food and water *ad libitum*. 10^6^ B16F10 cells in 200 μl PBS were injected subcutaneously into the back of the mice. RNAi experiments were performed 10 days after the injection of the tumour cells (day 0) as previously described 30. At day 0 and day 3, 12 μg of CTRL siRNA or AQP1 siRNA were complexed with 50 μl of oligofectamine (www.thermofisher.com) in a total volume of 200 μl of 5% glucose in water. After 20 min of incubation, the siRNA‐oligofectamine complexes were injected into the tumour using an insulin syringe with a permanently attached needle. At day 6, 24 μg of siRNA was used, and the amount of oligofectamine was scaled up accordingly. Tumour length (L) and width (W) were measured every 3 days after implantation using a caliper, and tumour volume was calculated as L × W^2^ × 0.5. Mouse survival was closely monitored (at least three times per day) throughout the entire experimental period. After death, tumour tissues were divided to be kept at −80°C and fixed in 4% paraformaldehyde for immunofluorescence analysis. Lungs were harvested and fixed in freshly prepared 4% paraformaldehyde for 4 hrs. Tumour foci on the surfaces of the lungs were counted under a stereomicroscope. Each organ was observed for metastasis analysis.

### gDNA analysis

Genomic DNA (gDNA) was extracted from frozen tumour tissue using the Qiagen DNeasy Blood and Tissue kit (www.qiagen.com) following the manufacturer's guidelines. The extracted gDNA was quantified using NanoDrop. For analysis, 3 μg of each sample was analysed by agarose gel electrophoresis.

### Antibodies

The following primary antibodies were used goat polyclonal anti‐actin (I‐19) (sc‐1616) dilution 1:500 and mouse monoclonal anti‐MMP2 (8B4) (sc‐13595) dilution 1:200 (www.scbt.it), rabbit polyclonal anti‐HIF‐1α‐CHIP grade (ab2185) 1:500 (www.abcam.com); rabbit polyclonal anti‐CASP3 (3015‐100) dilution 1:100 (www.biovison.com). The incubation time was O/N at 4°C for Western blot and 1 hr at RT for immunofluorescence analysis. The following secondary antibodies were used for Western blot: horseradish peroxidase (HRP) conjugated goat anti‐rabbit IgG (sc‐2004), and goat antimouse IgG (sc‐2005), donkey anti‐goat IgG (sc‐2020) dilution 1:5000 (www.scbt.it). The secondary antibody used for immunofluorescence analysis was a donkey anti‐rabbit Alexa 488‐conjugated (A21206) dilution 1:1000 (www.thermofisher.com).

### Protein sample preparation

Excised tumours were solubilized in at least seven volumes of RIPA buffer (25 mM Tris–HCl, pH 7.6; 150 mM NaCl; 1% Triton X‐100; 1% sodium deoxycholate; 0.1% SDS) added with a cocktail of protease inhibitors (www.lifescience.roche.com). The lysis was performed on ice for 1 hr, and the samples were then centrifuged at 22,000 × *g* for 1 hr. The protein content of the supernatant was measured with a bicinchoninic acid (BCA) Protein Assay Kit (www.Thermoscientific.com/Pierce).

### Western blot analysis

Western blot analysis was performed as previously described 33. Equal amounts of protein sample were separated by 8%, 10% and 15% Tris‐Glycine‐SDS‐PAGE gel (www.Thermoscientific.com/NuPage) and transferred to polyvinylidene difluoride membranes (www.merckmillipore.com). Membranes with blotted proteins were incubated with primary antibodies, washed, and incubated with peroxidase‐conjugated secondary antibodies. Reactive proteins were revealed with an enhanced chemiluminescent detection system (www.Thermoscientific.com) and visualized on a Chemidoc imaging system (www.bio-rad.com). Densitometric analysis was performed with ImageJ software (www.imagej.nih.gov).

### Immunofluorescence analysis

Eight micrometre transverse sections were prepared using a cryostat (www.leica-microsystems.com/) and stored on positively charged glass slides (www.Thermoscientific.com). The sections were fixed in 4% paraformaldehyde, washed with PBS, blocked using 0.1% gelatin diluted in PBS for 30 min at room temperature (RT), incubated for 1 hr with primary antibodies, washed with PBS‐gelatin, incubated with Alexa Fluor488 conjugated secondary antibody and mounted with a medium containing 50% Glycerol and 1% *n*‐propylgallate in PBS. Sections were examined with a Leica DMRXA photomicroscope equipped for epifluorescence and PL Fluotar 16× and 100× objectives (www.leica-microsystems.com/). Digital images were obtained with a DX M1200 digital camera (www.nikoninstruments.com) and ACT‐1 version 2.20 software (www.nikoninstruments.com). Once captured, the auto contrast function was applied to the whole images using Adobe Photoshop CC 2014 (www.adobe.com). Mean fluorescence intensity analysis was performed with ImageJ (www.imagej.nih.gov): 10 fields for each condition were converted in grey scale, and the mean grey value was obtained for each whole image.

### Statistics

A Kaplan–Meier plot was generated to determine mouse survival. Significance of differences in survival was calculated using Log‐rank (Mantel‐Cox) test. Statistically significant differences in number of lung nodules and densitometric analysis were computed using the Student's *t*‐test, the level being set at *P* < 0.05. Statistically significant differences in tumour growth were computed using one‐way ANOVA, the level being set at *P* < 0.05. All statistical analysis was performed with GraphPad Prism version 6.00 (www.graphpad.com/scientific-software/prism). Data are expressed as mean ± S.E.M.

## Results

### Intratumoural silencing of AQP1 expression prolongs survival time in the mouse model of melanoma

**Figure 1:**
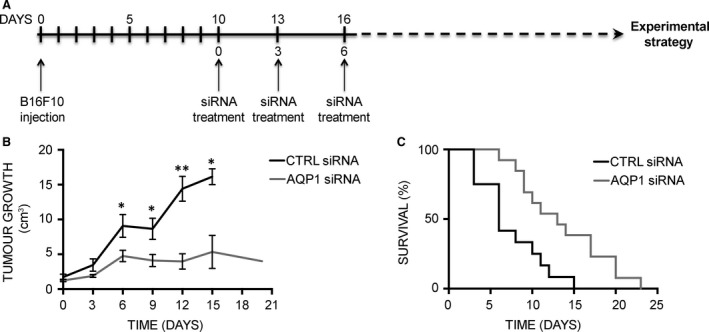
AQP1 silencing prolongs survival in the mouse model of melanoma. (**A**) Schematic representation of mouse treatment strategy for the analysis of tumour growth and animal survival. (**B**) Effect of AQP1 silencing on subcutaneous tumour growth. The diagram shows tumour growth of CTRL group (black line, *n* = 8) *versus* AQP1 siRNA‐treated group (grey line, *n* = 8). Data are expressed as the means ± S.E.M. (*P < 0.05, **P < 0.005). (**C**) Kaplan–Meier plot of CTRL siRNA‐ (black line, *n* = 12) *versus* AQP1 siRNA‐treated group (grey line, *n* = 13).

We have previously demonstrated that AQP1 silencing by RNA interference (RNAi) reduced primary tumour growth in the mouse model of melanoma 30. The first set of RNAi experiments was here performed to verify the involvement of AQP1 reduced expression in survival time (Fig. 1). The specific experimental strategy is shown in Figure 1A: B16F10 cells were injected into the flank of C57BL6/J mice and a visible tumour developed at the injection sites after 10 days. siRNAs (AQP1 siRNA or CTRL siRNA) were delivered by three intratumoural injections performed after 10 (day 0), 13 (day 3) and 16 (day 6) days. As untreated and CTRL siRNA‐treated mice showed similar growth in our previous study 30, the untreated group was omitted.

Starting from day 0, tumours were measured every 3 days to monitor tumour growth that, in line with previous experiments, was found significantly reduced in AQP1 siRNA compared with CTRL siRNA and untreated mice (Fig. 1B). AQP1 siRNA‐ and CTRL siRNA‐treated mice were therefore compared for their lifespan. The mouse survival over time is shown in a Kaplan–Maier plot (Fig. 1C). AQP1siRNA‐treated mice showed a twofold survival advantage compared to mice receiving CTRL siRNA, with a median survival time of 13 days compared to 6 days. These results indicate that intratumoural silencing of AQP1 expression prolongs survival time in mice.

### Intratumoural silencing of AQP1 expression strongly impairs metastatic lung nodule formation in the mouse model of melanoma

**Figure 2:**
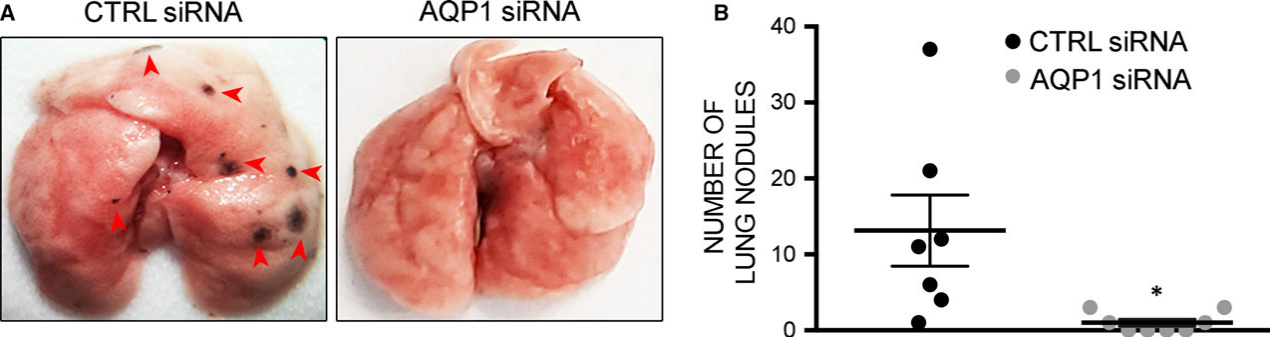
AQP1 silencing impairs metastatic lung nodule formation. (**A**) Representative images of lungs from CTRL and AQP1 siRNA‐treated mice after subcutaneous injection of B16F10. (**B**) Number of metastases counted at the day of death (in the *x*‐axis) in the lung from CTRL (*n* = 7) and AQP1 (*n* = 8) siRNA‐treated mice. Data are expressed as the means ± S.E.M. (*P < 0.05).

Resulting tumours and lungs were explanted immediately after the mouse death to analyse tissue morphology and biochemistry. Tumour colonies on all five lobes of the harvested lungs were counted macroscopically (Fig. 2A). The quantitative analysis, shown in Figure 2B, revealed that metastatic colonization of the lungs was significantly reduced in AQP1 siRNA‐treated group (1 ± 0.46 nodule/lung) compared with CTRL siRNA‐treated group (13.14 ± 4.67 nodule/lung). However, the number of metastasis did not correlate with survival time for CTRL siRNA‐treated tumours (data not shown) indicating that lung nodule formation is not the cause of mouse death in this mouse model which is instead due to extensive haemorrhage from ulceration of tumours 34. These results indicate that AQP1 blockade almost fully prevents lung metastases but does not prevent animal death caused by tumour ulceration still occurring, although delayed, in AQP1 siRNA‐treated tumours.

### Reduced AQP1‐dependent angiogenesis caused hypoxia and tumour cell apoptosis

**Figure 3:**
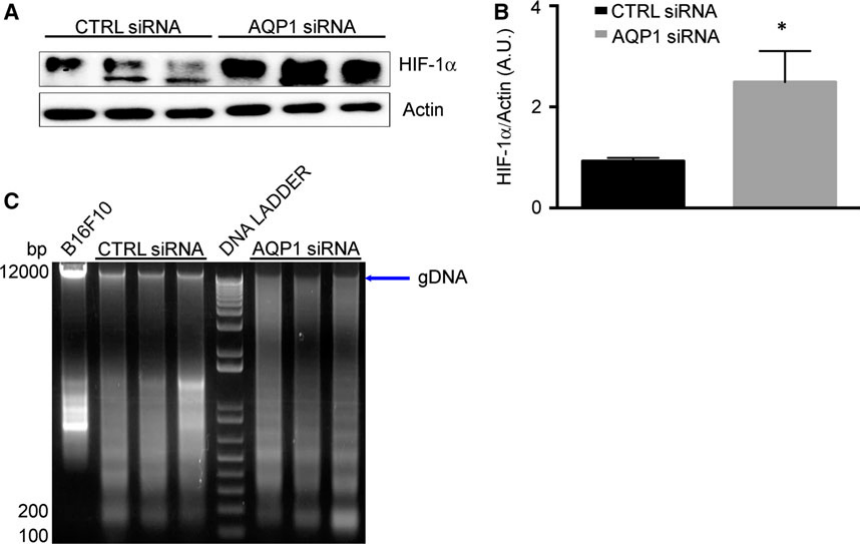
AQP1 silencing causes hypoxia and DNA degradation. (**A**) Immunoblot analysis of HIF‐1α expression in tumour explanted from CTRL siRNA‐ and AQP1 siRNA‐treated mice, as indicated. Actin was used as internal control for protein concentration. (**B**) Bar chart showing the densitometric analysis of HIF‐1α expression in CTRL (*n* = 3) and AQP1 (*n* = 3) siRNA‐treated mice. Data are expressed as the means ± S.E.M. (*P < 0.05). (**C**) Agarose gel electrophoresis showing the integrity of genomic DNA (gDNA, arrow) extract from tumours of CTRLs (*n* = 3) and AQP1 (*n* = 3) siRNA‐treated mice. Lane DNA ladder contains a 100‐bp marker.

**Figure 4:**
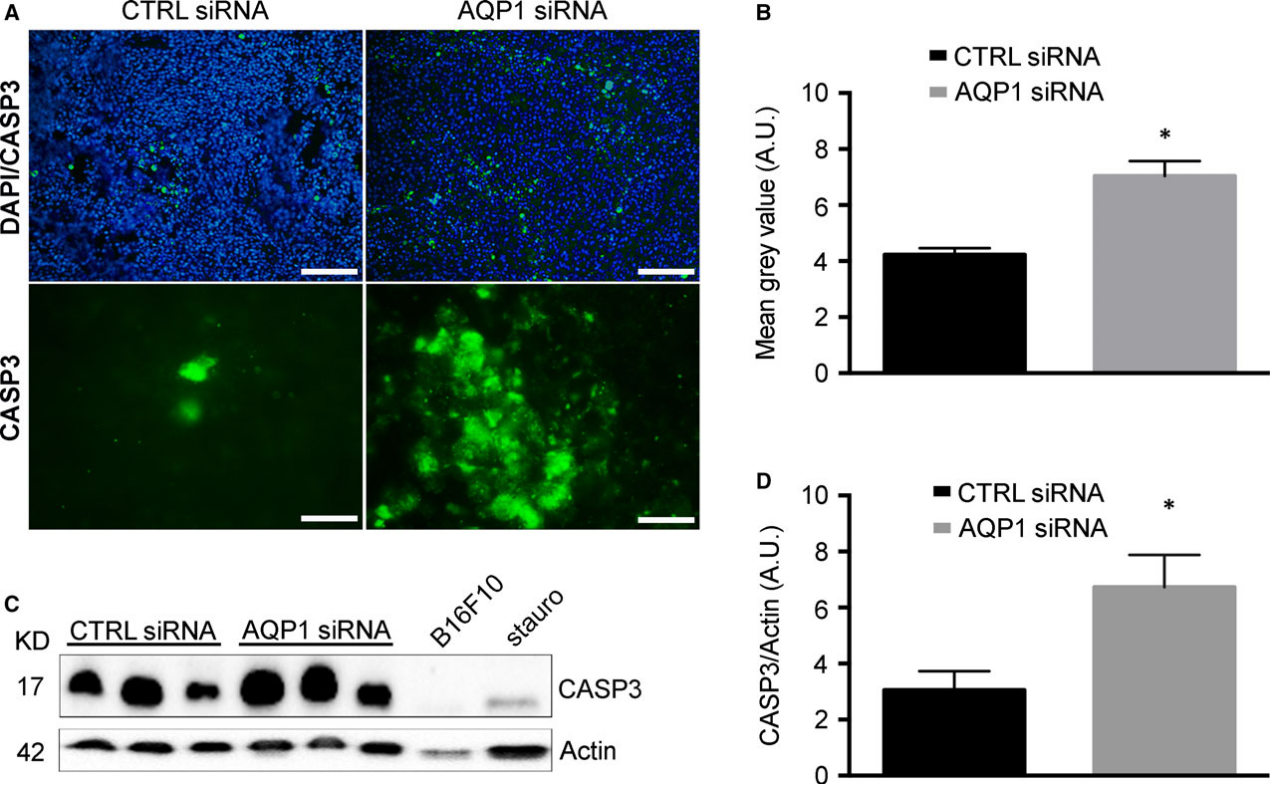
AQP1 silencing causes increased caspase activity. (**A**) Immunofluorescence analysis performed on tumour cryosections with antibodies against CASP3 (green) at lower magnification (top, scale bar 100 μm) and with DAPI staining of nuclei (blue) and at higher magnification (bottom, scale bar 20 μm). (**B**) Quantitative analysis of immunofluorescence experiments shown in (**A**). The bar chart shows the fluorescence intensity of CASP3 staining performed on whole images converted in grey scale. Data are expressed as the means ± S.E.M. (*P < 0.05). (**C**) Immunoblot analysis of cleaved CASP3 expression in tumours explanted from CTRL siRNA‐ and AQP1 siRNA‐treated mice, as indicated. Actin was used as internal control for protein concentration. B16F10 cells treated with 2 μM of staurosporine (stauro) for 48 hrs were used as positive control for CASP3 activation; untreated B16F10 cells were used as negative control. (**D**) Bar chart showing the densitometry analysis of CASP3 expression in CTRL (*n* = 5) and AQP1 (*n* = 5) siRNA‐treated mice. Data are expressed as the means ± S.E.M. (*P < 0.05).

The analysis was later focused on testing whether the reduction in AQP1‐dependent vascularization caused hypoxia and tumour cell apoptosis. We first analysed the expression level of HIF‐1α (Fig. 3 and B), which resulted up‐regulated in AQP1 siRNA‐treated tumour, as expected, being AQP1 silencing associated with reduced tumour angiogenesis 30. Next, two analyses were performed to look for cell apoptosis, the DNA fragmentation assay and the quantification of active CASP3. The integrity of gDNA, extracted from harvested tumour, was analysed by agarose gel (Fig. 3C). AQP1 siRNA‐treated tumours exhibited decrease in high molecular weight DNA compared to that of negative control (untreated B16F10 cells) and CTRL siRNA‐treated tumours. The CASP3 was analysed by immunofluorescence (Fig. 4A and B) and by Western blot (Fig. 4C and D). Immunofluorescence experiments performed on tumour cryosections showed a significantly higher CASP3 staining for AQP1 siRNA‐treated compared to CTRL siRNA‐treated tumours as confirmed by fluorescence intensity analysis quantification. This result was also confirmed by the analysis of cleaved CASP3 protein expression levels showing a twofold higher expression in the tumours treated with AQP1 siRNA‐compared to the CTRL siRNA‐treated. These results, in agreement with DNA fragmentation analysis, suggest that reduced AQP1‐dependent vascularization induces tumour cell apoptosis.

### Reduced AQP1‐dependent tumour angiogenesis induces a reduction in MMP2 expression

**Figure 5:**
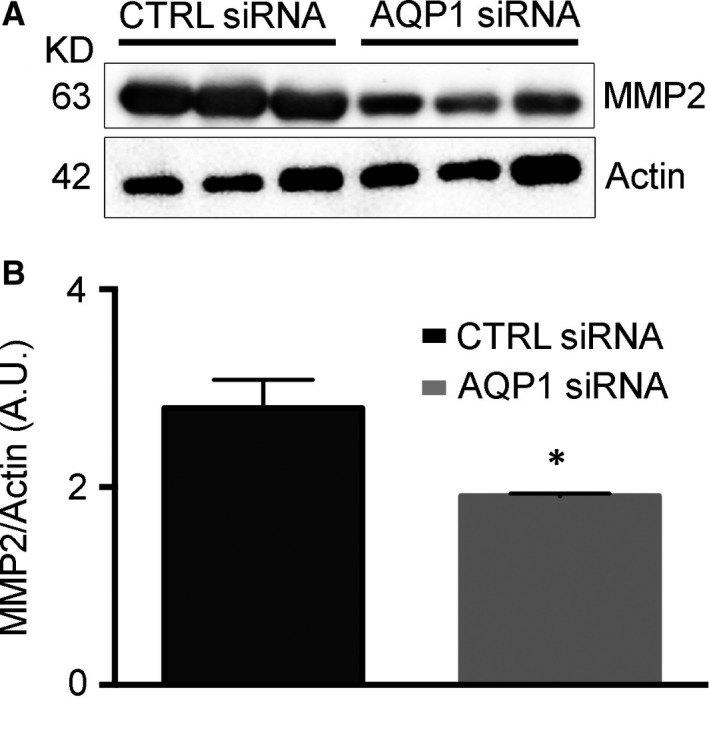
Metalloproteinase‐2 expression is reduced in AQP1 siRNA‐treated tumours. (**A**) Western blot analysis revealing a reduced MMP2 expression in AQP1 siRNA‐compared with CTRL siRNA‐treated tumours. Actin was used as internal control for protein concentration. (**B**) Bar chart showing the densitometric analysis of MMP2 expression in CTRL (*n* = 5) and AQP1 siRNA samples (*n* = 5). Data are expressed as the means ± S.E.M. (*P < 0.05).

We finally wanted to analyse the expression levels of proteins strictly involved in tumour progression, metastases and ECM remodelling, such as MMPs (Fig. 5). In particular, MMP2 activity plays an important role in endothelial cell migration, a key feature of tumour angiogenesis. The analysis was performed by Western blot and densitometric analysis (Fig. 5A and B). Results revealed a reduced expression of MMP2 in AQP1 siRNA‐treated tumours compared to CTRLs, indicating that reduced AQP1‐dependent vascularization is also cause of MMP2 reduced expression.

## Discussion

**Figure 6:**
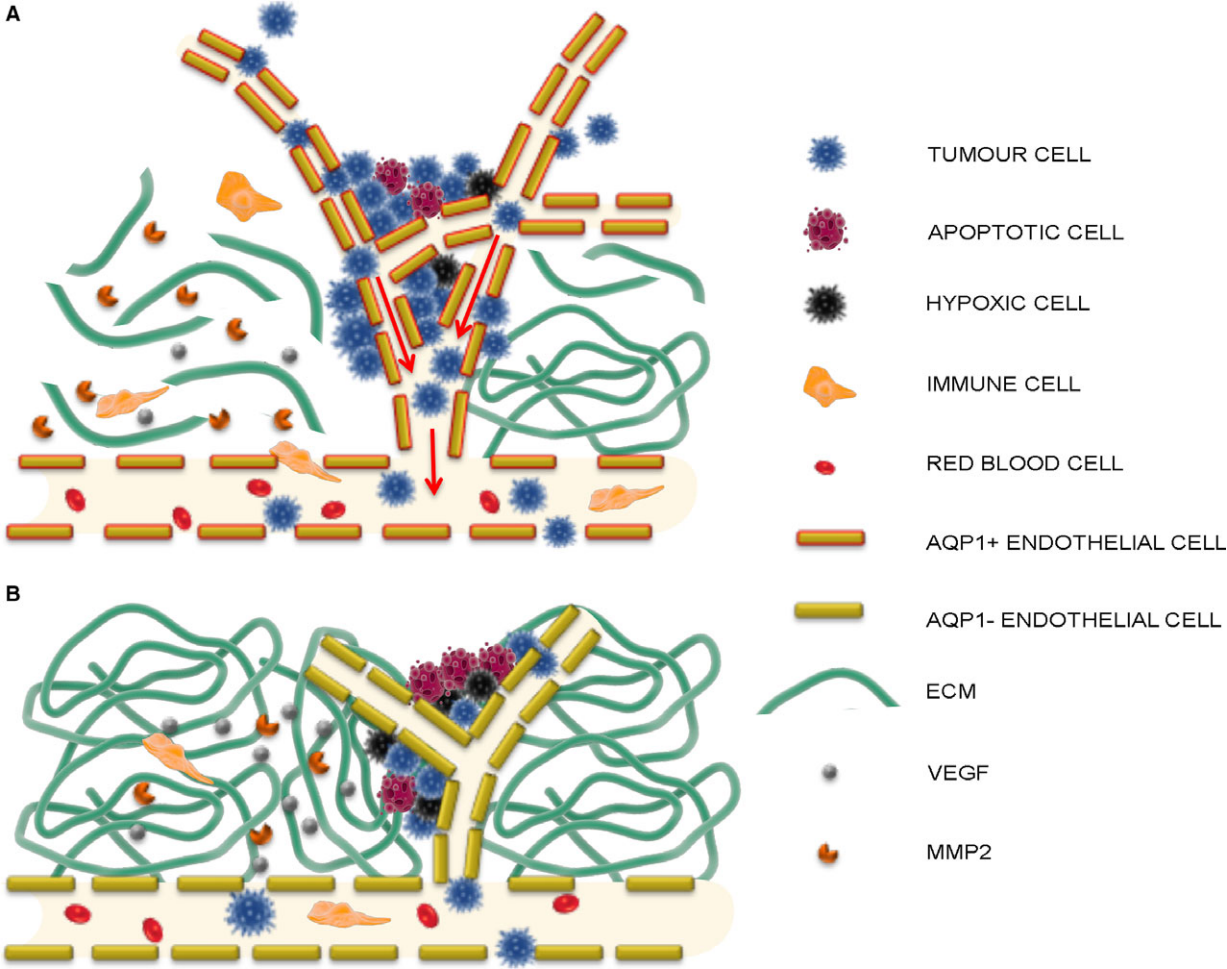
Schematic overview of AQP1‐dependent tumour angiogenesis and tumour cell intravasation. Summary of the suggested mechanism of AQP1 role in tumour angiogenesis based on the data here obtained and the previous studies by Saadoun *et al*. 11 and Nicchia *et al*. 30. (**A**) In control condition, the growing tumour cells secrete VEGF responsible for neovessel formation which is faster in AQP1 positive migrating endothelial cells (AQP1+ endothelial cells) 11 and is facilitated by the digestion activity of MMP2 against the extracellular matrix (ECM). MMP2 is also responsible for VEGF proteolytic release from the tumour matrix 26. Besides being secreted by resident endothelial and tumour cells, MMP2 is also secreted by immune cells extravasating from the vascular bed due to the higher level of permeability of the newly formed tumour vessels 38. The higher level of permeability of tumour vessels is also the route for tumour cells to escape the tumour (intravasation, arrows) and form metastasis elsewhere. (**B**) AQP1 endothelial cell inhibition (AQP1− endothelial cells) in AQP1 siRNA‐treated tumours is responsible for reduced endothelial cell migration, impaired neovessel formation and tumour growth 11, 30. The reduced number of MMP2 secreting tumour and endothelial cells itself causes either a reduced level of ECM digestion, further obstructing neovessel formation or a reduced release of VEGF from the ECM. As a result, AQP1 siRNA‐treated tumours are characterized by hypoxic conditions (hypoxic cells) causing cell apoptosis (apoptotic cell). Moreover, the impaired angiogenesis decreases the possibility either for immune cells to extravasate in the tumour or for tumour cell to intravasate and form metastasis in other organs and tissues. A reduced endothelial permeability of AQP1‐endothelium could enhance both the decreased extravasation of immune cells and intravasation of tumour cells.

The key finding of the present study is that endothelial AQP1 inhibition is able to reduce metastatic formations in the lung of a well‐known mouse model of melanoma 11, 35. Trying to get inside to the mechanism responsible for the reduced tumour growth and reduced metastatic formation, we have found that reduced AQP1‐dependent neovessel formation is responsible for HIF‐1α increase, reduced expression of MMP2 and increased expression of caspase‐3. Based on these data, and considering the previously demonstrated increase in VEGF expression in AQP1 siRNA‐treated tumours 30, we here propose a mechanism (Fig. 6) in which the reduced neovessel formation in AQP1 silenced tumours is responsible for hypoxia, documented by HIF‐1α increase, in turn causing VEGF increase, as expected. However, HIF‐1α dependent VEGF increase would not be able to induce the same level of tumour angiogenesis as that found in control tumours and this could be due to the slower cell migration of AQP1 silenced endothelial cells 11.

Based on the available literature in fact, the effect of AQP1 inhibition on tumour growth seems to be linked to the physiological role of AQPs in cell migration reported to have key pathophysiological effects on two dissimilar conditions apparently very different from each other: tumour angiogenesis and glial scar formation. It is intriguing that even if they occur in completely different pathological scenarios, in both cases AQPs expression would facilitate the cell volume regulation mechanisms as well as calcium signalling, both of which are necessary for migration to occur 5. In both cases, migration takes place thanks to a signalling molecule (such as VEGF for angiogenesis or inflammatory cytokines for glial scar formation) able to activate and attract the cells (endothelial cells or astrocytes) towards the site where their pathophysiological contribution is needed. As a consequence, activated endothelial cells can form new vessels feeding the tumour, while activated astrocytes can heal the wound forming the scar around the injured CNS area. There is abundant literature showing that the activation of the endothelial cell (or astrocyte), is associated with up‐regulation of AQPs and that AQP deletion or inhibition is able to inhibit tumour angiogenesis or glial scar formation, most likely by acting on the speed of cell migration 9, 10, 12.

Based on our data, however, AQP1‐dependent reduced angiogenesis could also be due to the reduced amount of MMP2 found in AQP1 siRNA‐treated tumours. MMP2 is responsible for extracellular matrix (ECM) digestion and VEGF proteolytic release from the tumour matrix 26, 36; therefore, the reduced expression of MMP2 found in AQP1 KD tumours might also reduce the amount of the available VEGF necessary to induce a normal level of tumour angiogenesis. This means that although under hypoxic condition VEGF is produced in AQP1 siRNA‐treated tumours, it remains trapped in the extracellular matrix (ECM), therefore reducing its effect in promoting angiogenesis.

This generates a persistent hypoxic condition, which is in turn responsible for the apoptosis of tumour cells and the reduced size of the tumours.

The increased expression of both VEGF and MMP2 is frequently synchronized by the common regulator HIF‐1α 37. However, in our study, we show a reduced expression of MMP2, which is the data that we believe to be central in this study. As MMP2 is normally produced by resident tumour cells, the reduced amount of MMP2 found in AQP1 siRNA‐treated tumours is first of all the consequence of the reduced number of tumour cells. Interestingly, MMP2 is delivered by tumour‐infiltrating immune cell 38, which are considered markers of higher endothelial permeability. In a previous study, we associated for the first time, the typical higher level of tumour endothelium permeability with the increased expression of AQP1 in multiple myeloma samples 23 and other authors have since approved the same view 14, 39. Moreover, pathological angiogenesis has always been associated with AQP1 endothelial overexpression, even in CNS endothelium, which normally does not express AQP1, for example during tumour brain gliomas 18 or retinal neoangiogenesis 40. Based on these observations, we can therefore speculate that, in the absence of AQP1, the endothelial permeability is lower with reduced lymphocyte infiltration and reduced MMP2 causing the effects explained above. More importantly, reduced AQP1‐dependent endothelial permeability would also be an obstacle for tumour cells to escape the tumour and disseminate to form metastasis. This relationship between AQP1 expression levels and endothelial permeability could find its rationale in the total absence of AQP1 in the CNS endothelial cells forming the blood–brain barrier whose level of permeability is the lowest in the organism.

In conclusion, this study shows that AQP1 is not only able to promote tumour angiogenesis but it can also facilitate tumour cell intravasation and metastatic formation, probably promoting an increase in endothelial permeability. We believe this study adds knowledge on the intricate role of AQP1 at the vascular level and in tumour biology, which is key in view of potential alternatives to the current antiangiogenic therapy mainly based on VEGF pathways.

## Author contributions

L.S., C.D.G., F.P., A.C. and M.G.M. performed the research, L.S., C.D.C. and G.P.N. analysed the data, G.P.N., A.F. and M.S. designed the research. G.P.N. wrote the manuscript with substantial critical revision from L.S., C.D.G., A.F. and M.S.

## Conflict of interest

The authors declare no conflict of interest.
